# Genetic Programming of Hypertension

**DOI:** 10.3389/fped.2017.00285

**Published:** 2018-01-22

**Authors:** Sun-Young Ahn, Charu Gupta

**Affiliations:** ^1^Department of Nephrology, Children’s National Health System, Washington, DC, United States; ^2^The George Washington University School of Medicine, Washington, DC, United States

**Keywords:** genetics, hypertension, children, epigenetics, pharmacogenomics, pediatrics

## Abstract

The heritability of hypertension (HTN) is widely recognized and as a result, extensive studies ranging from genetic linkage analyses to genome-wide association studies are actively ongoing to elucidate the etiology of both monogenic and polygenic forms of HTN. Due to the complex nature of essential HTN, however, single genes affecting blood pressure (BP) variability remain difficult to isolate and identify and have rendered the development of single-gene targeted therapies challenging. The roles of other causative factors in modulating BP, such as gene–environment interactions and epigenetic factors, are increasingly being brought to the forefront. In this review, we discuss the various monogenic HTN syndromes and corresponding pathophysiologic mechanisms, the different methodologies employed in genetic studies of essential HTN, the mechanisms for epigenetic modulation of essential HTN, pharmacogenomics and HTN, and finally, recent advances in genetic studies of essential HTN in the pediatric population.

## Introduction

Hypertension (HTN) is a serious public health issue affecting both children and adults. Between 2009 and 2012, approximately 32.6% of adults in the US were reported to have HTN (1). In children and adolescents between 3 and 18 years of age, the prevalence of HTN has been reported to be 3.6% (2). Morbidity and mortality from HTN continue to be high in adults, with HTN accounting for an estimated 45% of deaths due to cardiac disease and 51% of deaths from strokes (3). Despite its widespread prevalence, however, the etiology of essential HTN remains largely unknown. A growing body of evidence supports the observation that HTN results from a complex interplay of genetic, epigenetic, and environmental factors. Genetic factors are thought to contribute to approximately 30–60% of blood pressure (BP) variation (3, 4). However, known genetic factors explain only 3% of BP variance (5), underscoring the fact that many genetic variants have yet to be discovered. Moreover, these findings suggest that other factors, such as gene–gene interactions and epigenetics, may play a vital role in the etiology of HTN.

The clinical implications for deciphering the genetic factors that contribute to variations in BP and response to antihypertensive medications are significant. Knowledge of an individual’s predisposition to HTN can help with early implementation of preventive measures and formulation of effective therapeutic plans. In addition, pharmacogenomic information can help with the selection of personalized medication regimens, which may optimize therapeutic responses and help to reduce health-care costs. In this review, we discuss the various forms of monogenic HTN, the different study designs used to investigate the genetic epidemiology of essential HTN, the epigenetics of essential HTN, HTN pharmacogenomics, and recent advances in the genetics of essential HTN in children.

## Monogenic HTN

**Figure 1:**
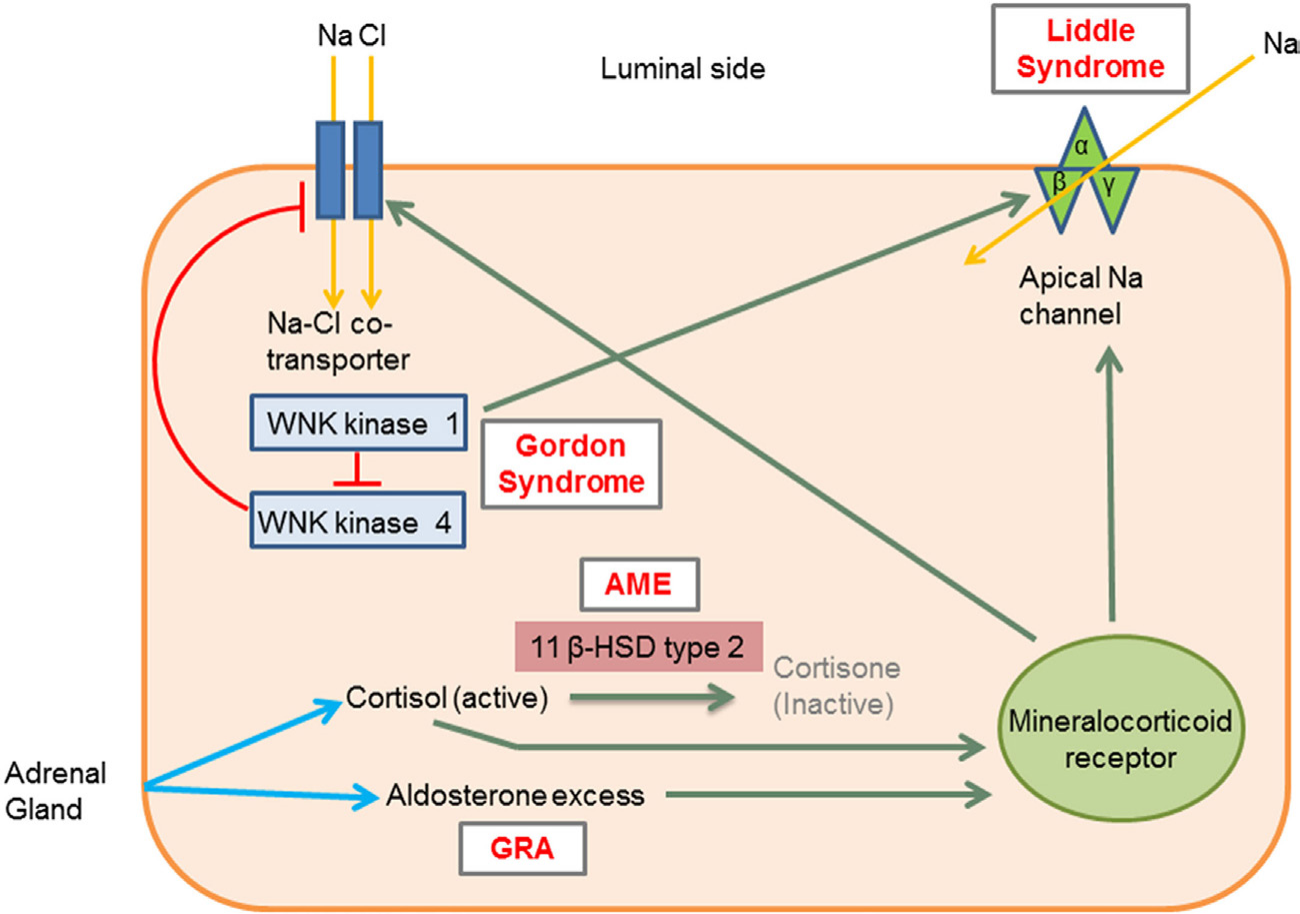
Molecular mechanisms involved in the different types of monogenic hypertension (HTN). Liddle syndrome: gain-of-function mutation in the gene encoding the apical epithelial sodium channel (ENaC) causes increased sodium absorption and subsequent HTN. Gordon syndrome: WNK1 normally inhibits WNK4, which in turn inhibits the Na–Cl cotransporter (NCC). WNK1 gain-of-function and WNK4 loss-of-function mutation increases the activity of the NCC leading to increased salt and water retention. AME: 11 β-hydroxysteroid dehydrogenase type II enzyme deficiency results in reduced cortisol conversion to cortisone (inactive form). Cortisol binds to the mineralocorticoid receptor and leads to signs of mineralocorticoid excess. GRA: a chimeric gene leads to excess aldosterone production, which acts on mineralocorticoid receptors. 11β HSD type II, 11 β-hydroxysteroid dehydrogenase type II enzyme; AME, apparent mineralocorticoid excess; GRA, glucocorticoid-remediable aldosteronism; Activation, green arrows; Inhibition, red lines with barheads. [Adapted from Simonetti et al. (18)].

Monogenic HTN syndromes refer to hypertensive disorders that follow Mendelian inheritance patterns due to single-gene mutations. Most monogenic forms of HTN are associated with volume expansion and low serum renin levels. A summary of the various types of monogenic HTN is provided in Table 1. Figure 1 presents the different pathophysiologic mechanisms that are involved in monogenic forms of HTN.

**Table 1:** Summary of the various forms of monogenic HTN.

	GRA	AME	CAH	Liddle	Gordon
Mode of inheritance	AD	AR	AR	AD	AD
Electrolyte abnormality	Hypokalemia/normal potassium Metabolic alkalosis	Hypokalemia/normal potassium Metabolic alkalosis	Hypokalemia/normal potassium	Hypokalemia/normal potassium	Hyperkalemia/normal potassium Mild metabolic acidosis
Time of onset of HTN	Early	Early onset for severe phenotype	Early	Early	Late
HTN severity	Moderate–severe	Moderate–severe	Severe	Moderate–severe	Severe
Aldosterone/renin level	Elevated aldosterone levels. Low renin and angiotensin II levels	Very low aldosterone and low renin levels	Low renin and aldosterone levels	Low renin and aldosterone levels	Aldosterone levels can vary. Low renin levels
Mechanism for HTN	Increased renal absorption of salt and water	Stimulation of MC receptor by cortisol	Excess cortisol precursors activate MC receptors	Increased renal absorption of salt and water	Increased Na–Cl cotransporter activity in the distal convoluted tubule
Genetic cause	*CYP11B1* gene fused with *CYP11B2* gene on chromosome 8q	Inactivating mutation in *HSD11B2* gene	Type IV: *CYP11B1* gene Type V CAH: *CYP17A1* gene	Mutation in *SCNN1B/SCNN1G* gene on chromosome 16p	*WNK 1* and *4* mutation (2 different loci on chromosome 12 and 17)
Other features	Cerebral hemorrhage Celtic ancestry	Similar presentation as licorice abuse	Type IV: ambiguous genitalia in girls, precocious puberty in boys Type V: primary amenorrhea in girls, ambiguous genitalia in boys		Hypercalciuria
Treatment	Glucocorticoids, amiloride, triamterene	Spironolactone, eplerenone, amiloride	Steroids, spironolactone, eplerenone	Low-salt diet. Triamterene or amiloride	Low-dose thiazides

*GRA, glucocorticoid-remediable aldosteronism; AME, apparent mineralocorticoid excess; CAH, congenital adrenal hyperplasia; AD, autosomal dominant; AR, autosomal recessive; MC, mineralocorticoid; HTN, hypertension*.

### Glucocorticoid-Remediable Aldosteronism (GRA)/Familial Hyperaldosteronism (FH) Type I

Glucocorticoid-remediable aldosteronism, an autosomal dominant disorder, was the first monogenic HTN syndrome to be identified (6). GRA is caused by a chimeric gene formed from the fusion of the promoter region of the 11 β-hydroxylase gene (*CYP11B1*) with the coding regions of the aldosterone synthase gene (*CYP11B2*) on chromosome 8q (7, 8). As a result of this chimeric gene, aldosterone production is activated by ACTH and becomes independent of renin regulation (7). The development of hyperaldosteronism, with resultant salt and water retention, leads to HTN. Patients with GRA typically present with mild hypokalemia, metabolic alkalosis, and low plasma renin levels. The early onset of GRA before 21 years of age and the development of significant hypokalemia with a thiazide diuretic are important clinical features of this condition (9).

Some patients with GRA may exhibit unique features such as cerebral aneurysms and intracranial bleeding. Therefore, screening by brain MRI at the onset of puberty in patients with GRA has been recommended (10). As the name suggests, GRA is remediable by glucocorticoids since they inhibit ACTH production, the stimulus for aldosterone production in GRA (11).

#### Other Rare Forms of FH

(i) FH type II: FH type II is characterized by the familial occurrence of aldosterone-producing adenomas or bilateral idiopathic adrenal hyperplasia that is unresponsive to glucocorticoids. This condition has a very similar clinical presentation to sporadic primary hyperaldosteronism (12); the only distinguishing feature is that a greater number of family members from the same kindred are affected by FH type II (13). The gene responsible for FH type II remains unknown and, therefore, diagnosis is usually challenging and based on exclusion of other conditions. Treatment of FH type II consists of administration of mineralocorticoid receptor antagonists and/or unilateral adrenalectomy for aldosterone-producing adenomas (14).
(ii) FH type III: The gene *KCNJ5* encodes an inward rectifier potassium channel Kir3.4. In FH type III, a gain-of-function mutation in the *KCNJ5* gene causes loss of membrane ion selectivity, triggering membrane depolarization and increased calcium entry into the adrenal glomerulosa cells. This in turn leads to hyperaldosteronism, HTN, adrenal hyperplasia, and severe hypokalemia (13, 15). Treatment usually requires bilateral adrenalectomy, especially in drug resistant cases.
(iii) FH type IV: discovered in five unrelated families by whole-exome sequencing, FH type IV is due to a gain-of-function mutation in the *CACNA1H* gene that encodes a T-type calcium channel (13). This mutated channel allows excess calcium entry into the adrenal glomerulosa cells and subsequent hyperaldosteronism (16). Mineralocorticoid receptor antagonists may be used for the treatment of FH type IV (14).

### Syndrome of Apparent Mineralocorticoid Excess (AME)

The syndrome of AME is an autosomal recessive disorder caused by an inactivating mutation in the *HSD11B2* gene, which encodes the 11β-hydroxysteroid dehydrogenase type II enzyme. This enzyme normally converts cortisol to the less active metabolite cortisone. With the inactivating mutation, excess cortisol accumulates and binds to the mineralocorticoid receptor, leading to symptoms of mineralocorticoid excess (17). Both mild and severe phenotypes of AME have been described. The mild AME phenotype manifests as mild HTN later in life with rare or no electrolyte abnormalities, while the severe phenotype presents early in life with severe HTN, failure to thrive, and early end organ damage (18). These phenotypic differences are likely related to differences in the level of enzyme expression. Whereas 11 β-hydroxysteroid dehydrogenase type II enzyme expression is almost absent in the severe phenotype of AME, it is present in varying degrees in the mild form of AME as a result of different mutations in the *HSD11B2* gene (19, 20).

Other clinical features of AME include hypokalemia with an increased trans-tubular potassium gradient, metabolic alkalosis, hypercalciuria, and nephrocalcinosis (18, 19). These clinical features are similar to those seen in licorice abuse, because licorice inhibits the same enzyme involved in AME. Genetic testing may be done to confirm the diagnosis. Treatment usually consists of mineralocorticoid receptor antagonists (spironolactone and eplerenone), epithelial Na channel blockers (amiloride), and thiazides (for hypercalciuria) with potassium supplementation as needed (18).

Geller syndrome, otherwise known as HTN exacerbated by pregnancy, is another mineralocorticoid excess syndrome caused by an activating mineralocorticoid receptor gene mutation. As a result of this mutation, the mineralocorticoid receptor loses its specificity for aldosterone and is activated by both aldosterone and progesterone. Inherited in an autosomal dominant manner, Geller syndrome leads to early HTN, which is exacerbated during pregnancy due to activation of the mineralocorticoid receptors by progesterone. Clinical features include normal serum potassium levels in the setting of low serum renin and aldosterone levels (21).

### Congenital Adrenal Hyperplasia (CAH)

Congenital adrenal hyperplasia results from defects in enzymes involved in cortisol synthesis (14). In type IV CAH (due to 11 β-hydroxylase deficiency) and type V CAH (due to 17 α-hydroxylase deficiency), the loss of cortisol feedback inhibition on the pituitary results in increased ACTH production and adrenal hyperplasia. This in turn leads to the accumulation of cortisol precursors, which cause increased salt and water uptake and subsequent HTN *via* activation of mineralocorticoid receptors. As a result, aldosterone production is suppressed (18).

Characteristic features of type IV CAH are precocious puberty, virilization due to excess sex hormone production with androgenic action, and early onset HTN (22). Type IV CAH is treated with steroids and mineralocorticoid receptor antagonists such as spironolactone for HTN.

Type V CAH has features opposite to type IV CAH due to sex hormone synthesis blockade, which manifests as delayed sexual development in girls and ambiguous genitalia in boys. Type V CAH is treated with steroids and sex hormones, in addition to mineralocorticoid receptor antagonists for HTN (18).

### Liddle Syndrome

Liddle syndrome is an autosomal dominant condition caused by a gain-of-function mutation in the *SCNN1B/SCNN1G* gene (located on chromosome 16p), which encodes the β and γ subunits of the epithelial sodium channel ENaC. This mutation causes an inability of ENaC to be removed from cell surfaces of the cortical collecting tubules, leading to increased sodium reabsorption and subsequent HTN (23). Patients with Liddle syndrome typically present with hypokalemia, metabolic alkalosis, low renin and aldosterone levels, and early onset HTN. Treatment includes a low salt diet and ENaC inhibitors, such as amiloride and triamterene (18).

### Pseudohypoaldosteronism Type II (Gordon Syndrome, Familial Hyperkalemic HTN)

Gordon syndrome is characterized by autosomal dominant inheritance of serine–threonine kinase gene (*WNK1* and *4*) mutations. Normally, WNK1 inhibits the function of WNK4, while WNK4 inhibits the expression of the Na–Cl cotransporter (NCC) (24). Therefore, a gain-of-function mutation in *WNK1* and loss-of-function mutation in *WNK4* collectively result in increased NCC expression and activity in the distal convoluted tubule (14). This leads to salt and water retention, followed by HTN (25). The increased salt reabsorption reduces sodium delivery to the cortical collecting duct, facilitating increased potassium absorption and hyperkalemia, which is typical of Gordon syndrome. ROMK channels, which aid in potassium excretion, can also be inhibited by the *WNK4* mutation, further causing hyperkalemia (8). Other metabolic abnormalities in Gordon syndrome include mild hyperchloremic metabolic acidosis, hypercalciuria, low urinary sodium excretion (26), low serum renin, and varying aldosterone levels. Metabolic abnormalities tend to occur earlier than HTN, which tends to present in adolescence or adulthood (27). Treatment of Gordon syndrome consists of low dose thiazide diuretics.

### HTN with Brachydactyly

Hypertension with brachydactyly is caused by a mutation in the *PDE3A* gene which encodes phosphodiesterase 3A (14). Patients affected by this syndrome have severe salt-independent HTN with short phalanges and metacarpals (28). The mechanism for HTN in this syndrome remains unknown, although it has been suggested that vascular smooth muscle cell hyperplasia and increased vascular resistance may play a role (28).

## Genetic Epidemiology Study Designs for Essential HTN

Traditional pedigree-based analyses are not very effective in genetic studies of essential HTN due to its complex nature. Therefore, other methodologies have been used to study the genetic epidemiology of essential HTN. The following section contains a brief description of the different study designs that have been employed in investigating the genetics of HTN, with a special focus on genome-wide association studies (GWAS) (7).

### Non-Parametric Linkage Analysis

Linkage refers to the tendency of two genes to be inherited together when they are in close physical proximity to each other on a chromosome (29). Based on this phenomenon, linkage analysis aims to locate the approximate position of a disease gene by using the location of a known marker gene (29, 30). The marker gene refers to a DNA sequence that has a known physical location and has a detectable phenotype. By investigating whether markers and disease traits co-segregate, linkage analysis can approximate the location of the disease gene (29). Non-parametric linkage analysis (or model-free analysis) is used when details regarding the disease, such as the genetic mode of inheritance, are not known (30). This method is particularly useful in studying complex diseases, such as essential HTN, where the mode of inheritance is unknown. Non-parametric linkage analysis of affected sibling pairs can provide significant insights into a particular HTN phenotype (7). However, a limitation of this method is that many affected sibling pairs are often required to achieve adequate power to detect statistically significant differences.

#### Discordant Sibling Pair Analysis

Discordant sibling pair analysis is a type of genetic linkage analysis that traces quantitative genetic trait loci. In this method, the square of the BP difference is measured as a function of the number of alleles that a sibling pair shares at known marker loci (31). If siblings with very discordant BPs are identified, then their genetic variation can be studied. The disadvantage of this method is that the process of identifying siblings with significant BP discordance can be quite challenging (7).

### Association Studies

Association studies are based on comparisons of a particular allele frequency between cases and unaffected controls/cohorts. These studies aim to determine whether an association is present between the particular allele and a disease trait (32). Association studies can be family-based or population-based (comprising unrelated individuals) and may use a case–control or cohort approach. Population-based studies are more widely used than family-based studies, since fewer resources are required to enroll cohorts than family-based studies. Population-based studies may also require less genotyping (33). One advantage of family-based association studies, however, includes protection against population substructure-related bias. This is a selection bias that occurs when study subjects come from population subgroups with different ancestries (34). This results in spurious differences in allele frequency between cases and controls/cohorts (35). In family-based association studies, study subjects within each family come from the same source population, minimizing selection bias. Another advantage of family-based association studies is the higher likelihood of true linkage and association when significant findings are identified (33).

### Genome-Wide Association Studies

Based on the concept of linkage disequilibrium at the population level, GWAS attempt to identify the association between genetic variants or single-nucleotide polymorphisms (SNPs), and common disease traits in populations (36). SNPs are located in particular genetic loci and refer to variations in single nucleotides (14, 37).

The Wellcome Trust Case Control Consortium (WTCCC) study, conducted in 2007, was the first study that attempted to identify variants associated with HTN using GWAS; however, no significant association was identified (38). Small sample size and the use of HTN as a discrete variable are some of the reasons for the failure of the WTCCC to identify an association between SNPs and BP (14, 39). The use of HTN as a discrete variable (presence or absence of HTN), as opposed to a continuous variable (systolic BP or diastolic BP), decreases study power and has therefore become an important consideration in subsequent GWAS designs (40).

In 2011, the International Consortium for BP GWAS identified 29 SNPs that were associated with HTN (41, 42). Since then, more than 60 SNPs have been identified that affect BP *via* mechanisms of sodium handling, kidney function, vasoconstriction, and molecular signaling (43–45). Examples of some novel SNPs linked to systolic BP and diastolic BP in both children and adults that have been identified through GWAS are listed in Table 2.

**Table 2:** Novel SNPs linked to elevated BPs identified through GWAS.

Locus	Lead SNP	Encoded protein function	Reference
*HIVEP3*	rs7515635	Modulates transcription	(46, 47)
*CSNK1G3*	rs6891344	Serine/threonine protein kinase involved in phosphorylation	(46, 48)
*PSMD5*	rs10760117	Subunit of ATP-dependent protease	(46, 49)
*MAP4*	rs319690	Involved in assembly of microtubules	(14, 50)
*MOV10*	rs2932538	Part of RNA helicase	(14, 51)
*ULK4*	rs3774372	Serine/threonine kinase	(14, 52)
*CSK*	rs1378942	Tyrosine kinase involved in actin remodeling	(53, 54)

*SNP, single-nucleotide polymorphism; GWAS, genome-wide association studies*.

Despite the identification of multiple SNPs associated with HTN, each of the common variants that have been discovered to this point appear to have only a small overall effect on BP (about 1 mmHg for systolic BP or 0.5 mmHg for diastolic BP) (41), with some rare variants noted to have a larger effect on BP (>1.5 mmHg) (55). These findings suggest that several genes may act in concert to modulate BP, and that other factors, such as gene–gene and gene–environment interactions, may contribute to BP variability.

A challenge of GWAS includes the difficulty in identifying the gene affected by the SNP, since the area of influence of the SNP may lie in distant genes (56). Some SNPs with genome-wide significance also exhibit pleiotropy and demonstrate strong independent links to more than one disease. For example, rs13333226 is independently associated with HTN and chronic kidney disease (57, 58).

Selection of cases and controls may also introduce a confounding bias in GWAS. False associations can be identified if the cases and controls are selected from different populations that have different baseline allele frequencies. This phenomenon is referred to as population stratification and may result when study subjects have different ancestries (35). Methods to address this issue include using genomic information to control for population structure, or using family-based study designs (29, 59). The selection of unaffected family members as controls in family-based study designs has the additional advantage of reducing environmental exposure confounders (60).

The recruitment of a large number of controls can be costly in GWAS due to the extent of genotyping involved. Thus, more studies are using genotypic information from subjects already enrolled as controls in other studies (60).

## Epigenetics of HTN

Epigenetic phenomena refer to changes in gene expression in the absence of alterations of the DNA sequence itself, and include posttranslational histone modification, DNA methylation, and non-coding microRNAs (miRNAs) (61). Although epigenetic modifications are heritable and can be passed on through several generations, they can also be influenced by nutritional, pharmaceutical, fetal, and environmental factors, and may be reversible. Epigenetic events play critical roles in physiological processes such as cellular differentiation, by ensuring that only certain genes are expressed in specific cell types (3). Abnormalities in epigenetic events can lead to the development of HTN, and in fact, HTN has been linked to several epigenetic phenomena as discussed below (62).

### DNA Methylation

DNA methylation involves the covalent binding of a methyl group to cytosine, forming 5-methylcytosine (5mC) within CpG dinucleotide sequences (61). The methyl groups come from S-adenosylmethionine, the availability of which is dependent on folate metabolism. This association with folate metabolism provides the basis for the strong link between DNA methylation and nutrition (61). DNA methylation of CpG dinucleotides (often located in the promoter regions) results in inhibition of transcription and therefore gene silencing (63). The onset and severity of HTN have been reported to be associated with the extent of DNA methylation (64). Smolarek et al. quantified the amount of 5mC in DNA from patients with essential HTN and found that lower levels of 5mC corresponded to higher stages of HTN (65). Lin et al. reported that hypomethylation of the angiotensin II type I receptor gene correlated with higher systolic and diastolic BPs. Smokers with HTN were also observed to have a lower level of methylation (66).

Interestingly, Meems et al. discovered that vitamin D-deficient parental rats had offspring with increased systolic and diastolic BPs (67). The offspring were found to have hypermethylation of the promoter region of the Panx1 gene. Furthermore, the offspring rats showed impaired endothelial relaxation, consistent with the fact that Panx1 encodes a hemichannel that plays a role in endothelial relaxation (67). These findings suggest that *in utero* nutritional status may affect childhood BPs; however, further research will be needed to determine whether prenatal and postnatal nutritional status have effects on the development of HTN in children (68).

### Histone Modification

Posttranslational modification of the N-terminal tail of histone proteins through processes such as methylation and acetylation can lead to changes in chromatin dynamics. This in turn leads to either decreased or increased gene expression (63). Both animal and human studies have shown associations between histone modifications and HTN. One such study reported that histone modifications resulted in angiotensin-converting enzyme 1 (ACE1) upregulation in organs from hypertensive rats (69). In human endothelial cells, cell-specific histone modifications were found to regulate mRNA levels of endothelial nitric-oxide synthase (70). Endothelial nitric-oxide synthase plays a role in BP regulation by modulating vascular tone through the production of nitric oxide in the vascular endothelium.

Interestingly, Wang et al. reported that ascorbic acid prevented the development of HTN in rat offspring prenatally exposed to lipopolysaccharide (LPS) (71). LPS exposure induced histone H3 acetylation in the ACE1 promoter region, resulting in increased ACE1 gene expression and HTN in rat offspring. Prenatal treatment with ascorbic acid, however, reversed the histone modification and led to less ACE1 gene expression (71). These findings suggest potential targets for novel antihypertensive therapies that can prevent or treat HTN early in life.

### Non-Coding RNAs

Non-coding RNAs are increasingly recognized as crucial regulators of gene expression and may influence cell-specificity of gene expression (61). Among non-coding RNAs, miRNAs have been the most widely studied in association with HTN. miRNAs are small non-coding RNAs, approximately 22 nucleotides in length, that silence mRNA expression through mRNA degradation or interference of mRNA translation (72). miRNAs have been reported to modulate BP through various mechanisms. One such mechanism is through the renin–angiotensin system pathway. In human kidneys, hsa-miR-663 was observed to regulate the mRNA levels of renin (*REN*) and apolipoprotein E (*APOE*) by binding to their 3′ untranslated regions (73). In addition, hsa-miR-181a was also found to regulate the mRNA expression of *REN* and apoptosis-inducing factor mitochondrion-associated 1 (*AIFM1*). Both miRNAs were downregulated in HTN, leading to increased expression of renin mRNA (73).

Studies are also ongoing for potential treatments for HTN based on epigenetic modifications. Mutations in mitochondrial DNA (mtDNA) have been linked to the development of HTN, proposedly through the action of reactive oxygen species (74). Consistent with these findings, Li et al. observed a decrease in mtDNA-encoded cytochrome *b* (mt-Cytb) and corresponding increase in reactive oxygen species in hypertensive rats (75). Interestingly, they found that when miR21, an miRNA that was found in higher levels in the hypertensive rats compared with controls, was injected into the hypertensive rats *via* a recombinant adeno-associated virus, there was an increase in mt-Cytb levels and lower BPs (75). The authors hypothesized that miR21 plays a compensatory role in HTN. Studies such as these are promising for the development of novel therapies that utilize epigenetic mechanisms, such as miRNAs, to treat HTN.

## Pharmacogenomics and HTN

Pharmacogenomics refers to the study of genes that can affect a patient’s response to drugs. The goal of pharmacogenomics is to develop tailored medications and doses that take into account the differences in each individual’s response to drugs. Extensive research has been performed on the genetic aspect of responses to antihypertensive medication, which include drug interaction with the target sites, drug transport, and metabolism. The Clinical Pharmacogenetics Implementation Consortium (CPIC), formed in 2009, establishes guidelines that aid with application of results from pharmacogenetic studies to actionable prescription of drugs (76). However, due to inconsistent results across studies and therefore insufficient evidence, there are no CPIC guidelines to date for antihypertensive medications (77, 78).

The International Consortium for Antihypertensive Pharmacogenomics Studies was established in 2012 to facilitate research of genetic variants that are responsible for interpatient variability in responses to antihypertensive medications (http://icaps-htn.org). To date, the most consistently reproducible pharmacogenomic data have been based on β-blockers and thiazide diuretics (78). Three genes, *ADRB1, NEDD4L*, and *YEATS4*, have been consistently linked with responses to antihypertensive drugs in various studies. The *ADRB1* gene encodes the β-1 adrenergic receptor, which is targeted by the β-blockers. Common SNPs in the *ADRB1* gene include the variants Ser49Gly (rs1801252) and Arg389Gly (rs1801253) (78). Patients who were homozygous for Arg389 and patients possessing the Ser49Arg389/Ser49Arg389 diplotype were reported to have a greater reduction in BP with metoprolol compared with those who were Gly allele carriers and those who had the Gly49Arg389/Ser49Gly389 diplotype, respectively (79, 80).

*NEDD4L* encodes a protein that downregulates the expression of ENaC in the distal nephron, thereby regulating sodium reabsorption in the kidneys (81). Several studies have shown that the more common G allele of rs4149601, located within the *NEDD4L* gene, is linked to greater systolic and diastolic BP reduction in response to thiazide diuretics (82, 83). These findings are consistent with the role of *NEDD4L* in reducing tubular sodium reabsorption.

Single-nucleotide polymorphisms (rs317689/rs315135/rs7297610) close to the *YEATS4* gene have also been associated with varying responses to thiazide diuretics (84). The *YEATS4* gene encodes a protein, GAS41, which is involved in regulation of cellular proliferation (78). Through GWAS, the rs317689/rs315135/rs7297610 haplotype was found to be significantly associated with diastolic BP response to hydrochlorothiazide (HCTZ) in African-Americans. The ATC haplotype was linked to a good response to HCTZ, while the ACT and the ATT haplotypes were associated with a poor response to HCTZ (84). The data on gene polymorphisms affecting responses to calcium channel blockers, ACE inhibitors, and angiotensin II receptor blockers are conflicting, and no candidate gene has shown consistent results (85, 86). A summary of recent pharmacogenomic findings on responses to antihypertensive medications is provided in Table 3.

**Table 3:** Genes associated with responses to antihypertensive medications [modified from Burrello et al. (14)].

Associated gene (single-nucleotide polymorphisms)	Antihypertensive drug response	Reference
*ADRB1* (rs1801252, rs1801253)	Greater response to metoprolol	Liu et al. (80); Johnson et al. (79)
*ADRB1* (rs 1801253)	Greater reduction in diastolic blood pressure (DBP) with carvedilol	Si et al. (87)
*ADRB2* (rs2053044)	Reached target mean arterial pressure faster with ramipril	Anthony et al. (88)
*NEDD4L* (rs4149601)	Greater systolic blood pressure (SBP) and DBP reduction in response to thiazide diuretics	Svensson-Färbom et al. (82); McDonough et al. (83)
*CAMK1D* (rs10752271)	Greater reduction in SBP in response to losartan	Frau et al. (89)
*YEATS4* (rs317689, rs315135, rs7297610)	ATC haplotype associated with greater reduction in DBP with thiazide diuretics	Turner et al. (84)

## Genetics of Essential HTN in Children

Pediatric genetic studies on HTN are scarce in comparison to adult studies and are often limited by small sample size. A recent study investigated the parental effects of 33 SNPs previously identified by GWAS on the BP of young offspring (53). Based on 1,525 subjects from the Family Atherosclerosis Monitoring In early life study, significant parental effects, albeit small, were reported for the SNPs rs11191548 (*CYP17A1*) and rs17367504 (*MTHFR*) (53). The paternal genotype of rs11191548 was found to be associated with elevated systolic and diastolic BP among offspring, whereas there was no association with the maternal genotype. Both the maternal and paternal genotypes of rs17367504 were associated with elevated systolic and diastolic BP among offspring. This study also observed that the SNP rs1378942 (*CSK*) demonstrated an association with systolic BP from birth to 5 years of age (53). *CSK* is a tyrosine kinase that plays a role in actin remodeling, which in turn has been shown to affect constriction of the arterial endothelium in murine newborns (54). Although limited by sample size, this was the first study to investigate the effect of parental SNPs on young offspring, and SNPs that affect BP in the early years of life.

In another study, the polymorphism T585C of the Y2 receptor (Y2R) gene was reported to be associated with systolic and diastolic BPs in obese children (90). Y2R is a receptor for neuropeptide Y, which is a potent constrictor of vascular smooth muscle cells. Y2R has also been observed to regulate neurogenic vasoconstriction in spontaneously hypertensive rats (91). Obese children homozygous for the T585 allele in *Y2R* showed significantly lower systolic and diastolic BPs compared with heterozygotes and C allele homozygotes (90).

Genetic predisposition for BP elevation spanning from childhood to adulthood was assessed in a longitudinal study that employed a combined genetic risk score formulated from 13 SNPs previously associated with HTN in adults (92). Subjects with a higher risk score at the age of 9 years had significantly higher diastolic BPs than subjects with a lower risk score. These subjects also had a higher risk for HTN in adulthood (92). Although the effect size was small (β = 0.68 mmHg) (92), this study provides a method for detecting individuals with a genetic predisposition for HTN early in childhood and may be used to identify those patients in which early preventive measures can be implemented.

The association between SNPs and BP in certain ethnic pediatric populations has also been reported in several recent studies. In a study of Chinese children, rs17249754 (*ATP2B1*) was found to be significantly associated with an increased risk for HTN (93). This polymorphism has also been previously linked to HTN in adults. *ATP2B1* encodes a calcium-transporting ATPase that modulates cellular calcium levels in the vascular endothelium, thereby regulating the contraction of vascular smooth muscle cells (94). In a study of Lithuanian children, the insertion/deletion (I/D) polymorphism (rs4340) for *ACE* was found to have a gender-specific association with BP (95). Boys with the *ACE I/D* and *ACE I/D* + *D/D* genotype had significantly increased odds for developing HTN (95), consistent with previous findings that adults homozygous for the D allele have higher plasma ACE concentrations than heterozygotes (96). Similar to these findings, the D-allele of the *ACE I/D* polymorphism was also associated with HTN in obese Brazilian boys (97).

Childhood HTN is a significant risk factor for HTN and cardiovascular disease in adulthood (98–100). Therefore, pediatric studies that identify genetic risk factors and modifiable epigenetic factors for HTN are further needed to formulate preventive strategies that can reduce childhood HTN, and therefore morbidity and mortality later in life. Moreover, drug pharmacokinetics differ between children and adults (101). Pediatric-based pharmacogenomic research would be beneficial in identifying the genes responsible for each child’s response to antihypertensive drugs. Antihypertensive drugs have multiple side effects that can have a negative impact on a child’s quality of life. Identifying the genes that predispose a child to poor or adverse drug responses would be beneficial in avoiding complications and optimizing therapeutic responses.

## Conclusion

Hypertension results from a complex interplay of genetic, epigenetic, and environmental factors. Due to this multifactorial interaction, elucidating single, specific genetic factors that contribute to the development of HTN has been challenging. Nevertheless, novel gene mutations and epigenetic factors causing BP variability continue to be discovered and have enhanced our understanding of BP modulation and the genetic programming of HTN. Interpatient variability in response to antihypertensive medication is well established, and the field of pharmacogenomics promises to provide guidelines for precision medicine and individually tailored antihypertensive regimens that would improve medication efficacy. The majority of genetic studies on HTN to date have been focused on adults, and there are currently few studies that have been conducted in the pediatric population. In view of the prevalence of HTN in the pediatric population, more studies on the genetic risk factors in this population are needed to enhance our understanding of the etiology of childhood HTN and to provide better preventive and therapeutic strategies for the future.

## Author Contributions

S-YA and CG contributed to the conception and writing of the manuscript. Both authors provided their final approval and agreed to be accountable for all aspects of the manuscript.

## Conflict of Interest Statement

The authors declare that the research was conducted in the absence of any commercial or financial relationships that could be construed as a potential conflict of interest.
